# *Trichoderma* Biofertilizer Links to Altered Soil Chemistry, Altered Microbial Communities, and Improved Grassland Biomass

**DOI:** 10.3389/fmicb.2018.00848

**Published:** 2018-04-30

**Authors:** Fengge Zhang, Yunqian Huo, Adam B. Cobb, Gongwen Luo, Jiqiong Zhou, Gaowen Yang, Gail W. T. Wilson, Yingjun Zhang

**Affiliations:** ^1^College of Agro-grassland Science, Nanjing Agricultural University, Nanjing, China; ^2^Department of Natural Resource Ecology and Management, Oklahoma State University, Stillwater, OK, United States; ^3^Jiangsu Provincial Key lab for Organization, National Engineering Research Center for Organic-based Fertilizers, Jiangsu Collaborative Innovation, Nanjing Agricultural University, Nanjing, China; ^4^Department of Grassland Science, China Agricultural University, Beijing, China

**Keywords:** *in situ* fertilization experiment, high-throughput sequencing, soil chemistry, key fungal genera, structure equation modeling

## Abstract

In grasslands, forage and livestock production results in soil nutrient deficits as grasslands typically receive no nutrient inputs, leading to a loss of grassland biomass. The application of mature compost has been shown to effectively increase grassland nutrient availability. However, research on fertilization regime influence and potential microbial ecological regulation mechanisms are rarely conducted in grassland soil. We conducted a two-year experiment in meadow steppe grasslands, focusing on above- and belowground consequences of organic or *Trichoderma* biofertilizer applications and potential soil microbial ecological mechanisms underlying soil chemistry and microbial community responses. Grassland biomass significantly (*p* = 0.019) increased following amendment with 9,000 kg ha^−1^ of *Trichoderma* biofertilizer (composted cattle manure + inoculum) compared with other assessed organic or biofertilizer rates, except for BOF3000 (fertilized with 3,000 kg ha^−1^ biofertilizer). This rate of *Trichoderma* biofertilizer treatment increased soil antifungal compounds that may suppress pathogenic fungi, potentially partially responsible for improved grassland biomass. Nonmetric multidimensional scaling (NMDS) revealed soil chemistry and fungal communities were all separated by different fertilization regime. *Trichoderma* biofertilizer (9,000 kg ha^−1^) increased relative abundances of *Archaeorhizomyces* and *Trichoderma* while decreasing *Ophiosphaerella*. *Trichoderma* can improve grassland biomass, while *Ophiosphaerella* has the opposite effect as it may secrete metabolites causing grass necrosis. Correlations between soil properties and microbial genera showed plant-available phosphorus may influence grassland biomass by increasing *Archaeorhizomyces* and *Trichoderma* while reducing *Ophiosphaerella*. According to our structural equation modeling (SEM), *Trichoderma* abundance was the primary contributor to aboveground grassland biomass. Our results suggest *Trichoderma* biofertilizer could be an important tool for management of soils and ultimately grassland plant biomass.

## Introduction

Grasslands are valuable ecological resources due to their biodiversity and ecosystem functions (Soussana et al., 2007; Hermann et al., 2016). In recent years, grasslands have been over-exploited for grazing with consequences such as soil carbon loss and desertification (Kosmas et al., 2014; Conant et al., 2017). Aboveground plant biomass is the foundation of grassland animal husbandry, which is often co-limited by both N and P in semi-arid grasslands (Zhan et al., 2017). Globally, many grasslands receive little nutrient input, and grassland fertility declines are linked to reduced grassland provisioning services, such as primary productivity (Sattari et al., 2016). Herbivore manure is important in grassland nutrient cycling; however, intensified livestock production can lead to excess manure. Deposition of this waste can contaminate groundwater and increase nitrate and phosphate concentrations, causing soil hardening and/or salinization, negatively impacting soil physicochemical properties and soil microbial communities (Groenigen et al., 2005; Sun et al., 2015; Xu et al., 2017). To mitigate these issues, manure can be processed into organic fertilizer via composting (Moral et al., 2009; Tang et al., 2011). When applied in grasslands, compost effectively increases soil fertility, supporting soil microbial communities and improving grassland productivity (van der Heijden et al., 2008; Wagg et al., 2014).

*Trichoderma* spp. are well-known plant growth-promoting fungi (Masunaka et al., 2011) that enhance plant nutrient uptake, production of growth hormones, and protect plants from pathogen infection (De Souza et al., 2008; Contreras-Cornejo et at., 2009; Zhang et al., 2013a). Many plant growth-promoting fungi are effective *in vitro*, but do not significantly benefit plants in field studies because of their inability to colonize root systems *in situ*. The survival rate and population density of plant growth-promoting fungi are prerequisites for their effectiveness in the rhizosphere (Raaijmakers et al., 2009; Zhang et al., 2013b). Formulation of inoculum carriers is necessary to ensure survival and maintenance of a viable *Trichoderma* population in field soils. Combination of organic fertilizers (compost) and *Trichoderma* strains as biofertilizers (BOF) may be an effective way to facilitate greater plant biomass compared to amendments of organic fertilizers or *Trichoderma* separately (Huang et al., 2011; Zhang et al., 2013a). Organic matter in BOF improves soil fertility while being an excellent substrate for the growth of *Trichoderma*. Zhang et al. (2013b) showed application of *Trichoderma* biofertilizer significantly altered fungal diversity and community in the rhizosphere of cucumber, resulting in improved yields. Additional researches demonstrated *Bacillus* and *Trichoderma* biofertilizers improved banana growth and increased soil microbial diversity (Shen et al., 2015; Fu et al., 2017; Xiong et al., 2017). However, to the best of our knowledge, microbial ecological mechanisms influencing soil microbial genera and subsequent stimulation of root exudation, which supports plant growth responses to biofertilizers are rarely researched in grasslands.

Rhizosphere chemistry is the sum of root exudates, their breakdown products, and the microbial products of soil-derived chemicals (Pierre et al., 2017). Based on this, we defined extracted chemicals in bulk soil as “soil chemistry.” Grasslands harbor diverse plant species with diverse root exudates. Soil chemistry composition plays an important role mediating plant and soil microbial interactions (Oburger and Schmidt, 2016). Dessaux et al. (2016) demonstrated plant roots convert their associated soil into complex mesotrophic environments, supporting highly diverse microbial communities. Root exudates provide a major food source for soil microbes and directly affect their assemblage and reproduction (Raaijmakers et al., 2009). The studies of Ng et al. (2014) and Salles et al. (2009) also reported soil carbon composition strongly influences soil microbial community composition. Hence, plant roots may drive multitrophic interactions in soil via root exudation. Furthermore, some chemicals with antifungal activities may produce a chemical barrier to pathogenic fungi (Yuan et al., 2012; Raza et al., 2015), thereby indirectly improving grassland biomass. Beneficial plant-microbe interactions have received less attention in grassland systems, but advances in microbial genetics are improving our ability to link key microbial genera with soil health and ecosystem productivity (Finkel et al., 2017).

In our study, we applied organic fertilizer (composted cattle manure) or *Trichoderma* biofertilizer (composted cattle manure + inoculum) at different rates (0 kg ha^−1^, 3,000 kg ha^−1^, 6,000 kg ha^−1^, or 9,000 kg ha^−1^) to meadow steppe grassland plots for two growing seasons. We focused on the potential microbial ecological mechanisms underlying observed grassland biomass. This investigation addresses the following questions: (i) How do different fertilization regimes impact grassland biomass? (ii) Does addition of *Trichoderma* further increase grassland biomass above the effects of cattle manure alone? (iii) Do different fertilization regimes shift soil chemistry and soil microbial communities? (iv) Is soil chemistry significantly correlated with specific microbial genera? (v) Are soil microbial diversities or communities significantly correlated with grassland plant biomass? (vi) How do different variables (i.e., key microbial genera, soil properties, soil chemistry, and soil microbial communities) contribute to grassland plant biomass? Our findings will provide a better understanding of above- and belowground responses to alternative grassland fertilization and may ultimately improve management of grassland soils for key provisioning services.

## Materials and methods

### *Trichoderma* strain, culture conditions, and conidia suspension

We utilized a strain of *Trichoderma rossicum*, NAU-18 (CCTCC No. AF2017008, China Center for Type Culture Collection), isolated from soil at the Grassland Agro-ecosystems Station (49°26′12″N, 120°8″52″E, 695 m altitude). NAU-18 was identified based on morphological characteristics and internal transcribed spacer (ITS) sequence analysis (Figures S1A,B). The strain was stored at −80°C in 30% glycerol before use and routinely cultured on potato dextrose agar (PDA) at 28°C. NAU-18 conidia suspension was prepared according to the procedure of Zhang et al. (2013a). The final concentration was 3.2 × 10^8^ colony-forming units (CFU) ml^−1^, based on hemocytometer counts. This conidia suspension was used as inoculum for solid-state fermentation.

### Preparation of biofertilizers (BOF)

The organic fertilizer (OF) used in our study was composted cattle manure, supplied by TeniheFarm (Hulunbuir, China). Cattle manure was composted at 30 to 70°C for ~20 days and contained 36.8% organic matter, 3.1% N, 2.5% P_2_O_5_, and 1.9% K_2_O, with moisture < 30%.

Wheat straw was utilized as a substrate for NAU-18 production. Sterile wheat straw and 1% urea were stirred thoroughly (moisture = 70%), after which NAU-18 conidia suspension was added to the substrate (9% of the total [v/w]), and solid-state fermentation was maintained at 30°C for 8 days in a shallow tray. The final dry weight density of NAU-18 was 1.1 × 10^10^ CFU g^−1^. Biofertilizer (BOF) was a mixture of 1:10 (w/w) *Trichoderma* NAU-18 fermentation products and composted cattle manure.

### Study site description and experimental design

Our *in situ* fertilization experiment was initiated in June 2015 at the Grassland Agro-ecosystems Station, located in northeastern Inner Mongolia, Hulunbuir, China. This region is characterized by a continental temperate semi-arid climate typically with 350–400 mm annual precipitation (Inner Mongolia Meteorological service, http://www.imwb.gov.cn). The growing season is from late June to early September with an average temperature range of 16–21°C. The soil in this region is classified as a chernozem (IUSS WG WRB, 2015) with pH 6.59, 44.5 g kg^−1^ soil organic matter, 189.3 mg kg^−1^ plant-available N, 3.3 mg kg^−1^ plant-available P, and 134.2 mg kg^−1^plant-available K. The grassland plant community is dominated by *Poapratensis* L., *Leymus chinensis* (Trin.) Tzvelev, *Bromusinermis* Leyss, *Stipabaicalensis* Rochev., and *Potentilla bifurca* L.

Two fertilizer types and three amendment rates were utilized, plus a non-amended control, resulting in seven treatments in a randomized block design with three replicates per treatment. Each grassland plot was 5 m long and 3 m wide, and the study was conducted for two growing seasons. The seven treatments were: (1) CK: non-amended; (2) OF 3,000: 3000 kg ha^−1^ organic fertilizer; (3) OF 6000: 6000 kg ha^−1^organic fertilizer; (4) OF 9000: 9,000 kg ha^−1^ organic fertilizer; (5) BOF 3000: 3,000 kg ha^−1^ *Trichoderma* biofertilizer; (6) BOF 6000: 6,000 kg ha^−1^ *Trichoderma* biofertilizer; and (7) BOF 9000: 9,000 kg ha^−1^ *Trichoderma* biofertilizer. All treatments were applied on July 10, 2015, and June 2, 2016. All experimental plots were completely dependent on precipitation during our study.

### Soil and vegetation sampling

Soil and vegetation samples were collected on August 25, 2016. Five cylindrical soil cores (0–10 cm depth, 6 cm diameter) were randomly collected and mixed to form a composite soil sample for each plot. All soil samples were sieved through 2.0-mm mesh and thoroughly homogenized. Each composite soil sample was then divided into three sub-samples: the first sub-sample was stored at 4°C for soil chemistry analysis, another was air dried at room temperature for 7 days for analysis of soil properties, and the final sub-sample was stored at −20°C for DNA extraction. Two random subplots (0.25 × 1.0 m) within each plot were destructively harvested for aboveground biomass. Shoots were cut at the soil surface and oven dried at 65°C for 72 h before weighing. Shoot dry weights were expressed as total grassland biomass per m^2^. Plant tissue crude protein was determined by Kjeldahl method (Da Silva et al., 2016).

### Extraction and identification of soil chemistry

For each replicate soil sub-sample, 5.0 g of soil were combined with 50 ml of ethyl acetate (1:10 ratio) in a 150 ml Erlenmeyer flask and shaken on a table agitator at 30°C for 2 h. The suspension was filtered through a 0.45-μm Millipore filter and concentrated to 0.5 ml at 35°C using a vacuum rotary evaporator (Yarong Model RE-52A, Shanghai, China). The concentrated solution analyzed by gas chromatography-mass spectrometry (GC-MS). The following protocol was used for GC-MS detection: 60°C for 3 min, followed by increasing the temperature at a rate of 5°C min^−1^ to 240°C, held at 240°C for 5 min, increased to 280°C at a rate of 20°C min^−1^, and held at 280°C for 2 additional minutes. The mass spectrometry detector (MSD) was operated in electron ionization mode at 70 eV, with a source temperature of 230°C. A continuous scan from 45 to 500 m/z was applied. Helium was the carrier gas at a linear velocity of 1.0 ml min^−1^. The chromatographic peaks of the components were compared with entries in the National Institute of Standards and Technology (NIST) database (Version 2.0) to characterize the variation in the chemical composition of soil sub-samples.

### Determination of soil physicochemical properties

Soil analyses were performed by the soil-testing lab in Qiyang at the red soil experimental station of the Chinese Academy of Agricultural Sciences. Soil pH was measured with soil-water (1:2.5, w/v) slurry using a compound electrode (PE-10; Sartorious, Germany). Soil organic C (SOC) and total N (TN) were determined with an Elementar Vario EL III (Germany). Plant-available N was hydrolyzed with 1.0 mol l^−1^ NaOH and measured according to the methods of Shi (1996). Total P was determined by perchloric acid digestion (Olsen and Sommers, 1982). Plant-available phosphorus (Olsen-P) was extracted with sodium bicarbonate and measured using the molybdenum-blue method (Watanabe and Olsen, 1965). Total and plant-available K were measured by flame photometry (Knudsen et al., 1982).

### DNA extraction, high-throughput sequencing, and bioinformatics

Total soil genomic DNA was extracted using a Power Soil DNA Isolation Kit (MoBio Laboratories Inc., Carlsbad, CA, USA) following the manufacturer's instructions. The concentration and quality (A260/A280 ratio) of the DNA samples were determined using a NanoDrop 2000 spectrophotometer (Thermo Scientific, Waltham, MA, USA). Pyrosequencing analyses of the 16S rRNA gene and ITS region were performed to determine the diversity and composition of bacterial and fungal communities, respectively. The V4 region of the bacterial 16S rRNA gene was amplified using the gene-specific primers 520F (5′-AYTGGGYDTAAAGNG-3′) and 802R (5′-TACNVGGGTATCTAATCC-3′), while the ITS1 region of the fungal ITS was targeted by the primers ITS1F (5′- CTTGGTCATTTAGAGGAAGTAA-3′) and ITS2 (5-GCTGCGTTCTTCATCGATGC-3). PCR amplification of the bacterial 16S rRNA and fungal ITS sequences was conducted in a volume of 30 μl containing15 μl of 2 × Master Mix (Thermo Scientific® Phusion High-Fidelity PCR Master Mix, New England Biolabs, UK), 0.5 μM each primer, 10 ng of soil DNA template and nuclease-free water to bring the total to 30 μl. The obtained PCR products were purified using a PCR Purification Kit (Axygen Bio, USA) and quantified with PicoGreen® dsDNA reagent (Promega, USA). The purified amplicons were then pooled in equimolar concentrations as a single aliquot and employed for library construction using the NEB Next® Ultra ™ DNA Library Prep Kit for Illumina (New England Biolabs, UK). All library preparation was performed on the Illumina MiSeq platform at Total Genomics Solutions Biotechnology Co., Ltd. (Shenzhen, China).

Raw sequences were assembled for each sample based on unique barcodes using Quantitative Insights Into Microbial Ecology (QIIME) after removal of the adaptor and primer sequences (Caporaso et al., 2011). Split sequences for each sample were merged using FLASH V1.2.7 (Magoc and Salzberg, 2011), and low-quality sequences were then discarded using QIIME. The sequences retained for each sample were analyzed following the UPARSE pipeline, using USEARCH and Perl scripts to generate an operational taxonomic units (OTUs) table and pick representative sequences (Edgar, 2013). Sequences with a quality score < 0.5 or a length < 200 nt and singletons were discarded. After that, retained sequences were assigned to OTUs at 97% similarity. We chose a representative sequence from each OTU, and the Ribosomal Database Project (RDP) classifier (the RDP Bacterial 16S database for 16S rRNA data and the UNITE Fungal ITS database for ITS data) was used to assign taxonomic information (Kõljalg et al., 2013). The MOTHUR (version 1.25.1) standard operating procedure (SOP) was employed for further analyses of pyrosequencing data (Wang et al., 2007; Schloss et al., 2009). To correct for sampling effects, randomly selected subsets of 27,942 sequences per sample for 16S and 30,629 sequences per sample for ITS were chosen for further bacterial and fungal community analyses.

### Statistical analyses and data accessibility

Statistical analyses of all parameters were performed using the IBM SPSS statistical software package version 20 (IBM Corporation, New York, USA). Data from each treatment were analyzed using one-way analysis of variance (ANOVA), and Duncan's multiple range tests (*p* < 0.05) were performed for multiple comparisons. The multiple comparison correction is according to (Benjamini and Hochberg, 1995) Non-metric multidimensional scaling (NMDS) plots and a principal component analysis (PCA) were performed in R with the vegan package (Version 3.0.2, Oksanen et al., 2009). Differences in soil chemistry and bacterial and fungal communities between treatments were tested by analysis of similarities (ANOSIM) (Yan et al., 2017).

SEM is a powerful tool to examine relationships among inter-correlated variables, in which the net effects of an experimental treatment can be partitioned into direct and indirect effects. Observed variables rather than latent variables were used in the models directly. In our study, SEM was employed to test the direct and indirect pathways between soil organic matter, total N, soil chemistry, bacterial and fungal communities, *Trichoderma* abundance, and aboveground grassland biomass, providing a comprehensive analysis of all relationships between factors. We performed SEM using AMOS software (IBM SPSS AMOS 20.0.0). The fit of the model was tested using the maximum likelihood (χ^2^) goodness-of-fit test with *p* < 0.05 along with root mean square error of approximation (RMSEA). Figures were generated with Sigmaplot 12.0 (Systat Software Inc., CA, USA). Raw bacterial 16S and fungal ITS sequence data are available at the National Center for Biotechnology Information (NCBI) under accession number PRJNA393964.

## Results

### Grassland biomass and crude protein

**Figure 1:**
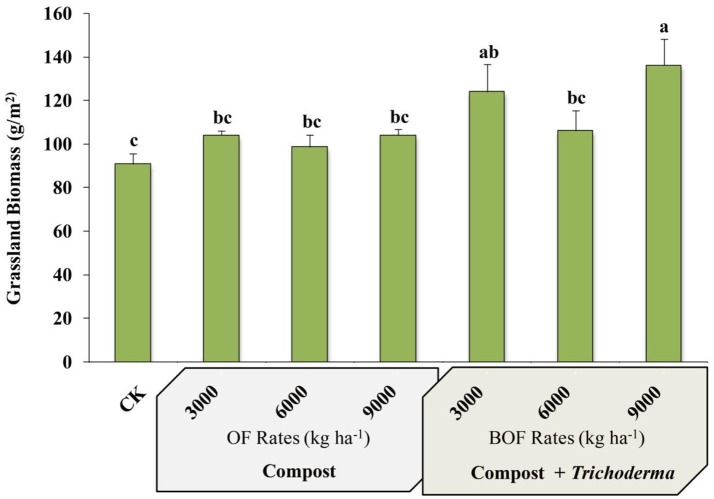
Grassland biomass by fertilization regime. CK: non-amended; OF 3000: 3,000 kg ha^−1^ organic fertilizer (composted cattle manure); OF 6000: 6,000 kg ha^−1^ organic fertilizer; OF 9000: 9,000 kg ha^−1^ organic fertilizer; BOF: 3,000 kg ha^−1^ biofertilizer (composted cattle manure + *Trichoderma* inoculum); BOF 6000: 6,000 kg ha^−1^ biofertilizer; BOF 9000: 9,000 kg ha^−1^ biofertilizer. Data from each treatment were analyzed using one-way analysis of variance (ANOVA). Bars represent mean values of three replicates ± SE. Bars that do not share a letter are significantly different (*p* < 0.05).

Plots fertilized with 9,000 kg ha^−1^ BOF had the greatest aboveground biomass of any treatment and were significantly more productive than non-amended plots and plots fertilized with any rate of OF or BOF, except for BOF3000 treatment (Figure 1). However, there was no significant difference in plant tissue crude protein between treatments or the CK, which was similar between all amended and CK plots (mean % ranged from 6.52 ± 0.27 to 7.31 ± 0.37).

### Soil chemistry and microbial communities

To identify potential soil microbial ecological mechanisms underlying observed differences in grassland biomass, we focused on three representative treatments (CK, OF 9000 and BOF 9,000, hereafter referred to as CK, OF, BOF). Ethyl acetate extracts from these soil samples were analyzed by GC-MS. The GC-MS analysis revealed 37 compounds, which showed significant (*p* < 0.05) differences in relative abundance between treatments (Table 1), including 20 alkanes, 3 benzenes, 4 alcohols, 1 acid, 1 ether, 2 aldehydes, 3 esters, 2 amines, and 1 phenol. The relative abundance of most compounds was significantly (*p* < 0.05) greater in plots amended with BOF, compared to non-amended control plots, and in several cases compared to OF plots.

**Table 1:** Relative abundances of identified soil chemistry by different fertilization regime.

**Category**	**ID#**	**Retention time**	**Name**	**CAS**	**Molecular formula**	**Peak areas (%)**		
						**CK**	**OF**	**BOF**
Alkanes	GC3	12.056	3-Methyl-5-propylnonane	31081-18-2	C_13_H_28_	0^b^	0.25 ± 0.06^a^	0.25 ± 0.01^a^
	GC6	13.33	Undecane, 3-methyl-	1002-43-3	C_12_H_26_	0.39 ± 0.12^b^	0.70 ± 0.19^ab^	1.17 ± 0.11^a^
	GC7	14.215	Pentadecane	629-62-9	C_15_H_32_	0.44 ± 0.13^b^	1.01 ± 0.05^a^	1.04 ± 0.02^a^
	GC8	14.567	Undecane, 2,5-dimethyl-	17301-22-3	C_13_H_28_	1.1 ± 0.26^b^	1.93 ± 0.10^ab^	2.65 ± 0.53^a^
	GC9	15.338	Nonadecylcyclohexane	22349-03-7	C_25_H_50_	0.64 ± 0.13^b^	1.25 ± 0.13^a^	1.65 ± 0.14^a^
	GC10	15.667	6-Methyldodecane	6044-71-9	C_13_H_28_	0.65 ± 0.08^a^	1.67 ± 0.12^a^	1.56 ± 0.08^a^
	GC11	15.717	5-(2-Methylpropyl)nonane	62185-53-9	C_13_H_28_	0.88 ± 0.20^a^	0^b^	0^b^
	GC16	17.051	Nonadecane	629-92-5	C_19_H_40_	1.22 ± 0.09^b^	1.72 ± 0.03^a^	1.86 ± 0.07^a^
	GC17	17.478	Pentadecane, 6-methyl	10105-38-1	C_16_H_34_	1.50 ± 0.08^b^	1.77 ± 0.41^b^	2.67 ± 0.05^a^
	GC20	18.21	1-Cyclohexylheptane	5617-41-4	C_13_H_26_	1.09 ± 0.06^b^	1.73 ± 0.04^a^	1.72 ± 0.02^a^
	GC24	18.736	7-Methylhexadecane	26730-20-1	C_17_H_36_	2.19 ± 0.08^a^	1.88 ± 0.05^b^	2.01 ± 0.03^ab^
	GC27	19.253	Cyclohexane,1,1,3-trimethyl-2-(3-methylpentyl)-	54965-05-8	C_15_H_30_	0.71 ± 0.04^b^	0.86 ± 0.05^a^	0.91 ± 0.07^a^
	GC28	19.409	Naphthalene,1,2,3,4-tetrahydro-6,7-dimethyl-	1076-61-5	C_12_H_16_	0.83 ± 0.02^b^	0.75 ± 0.04^b^	1.85 ± 0.13^a^
	GC30	20.039	Hexadecane	544-76-3	C_16_H_34_	0.57 ± 0.05^b^	0.63 ± 0.03^b^	1.17 ± 0.17^a^
	GC31	20.346	Decahydro-4,4,8,9,10-pentamethylnaphthalene	80655-44-3	C_15_H_28_	0.85 ± 0.07^b^	0.90 ± 0.11^b^	1.38 ± 0.14^a^
	GC32	20.596	Tetracontane,3,5,24-trimethyl-	55162-61-3	C_43_H_88_	0.64 ± 0.13^b^	0.68 ± 0.04^b^	1.21 ± 0.12^a^
	GC37	21.695	Hexadecane	544-76-3	C_16_H_34_	0.51 ± 0.06^b^	0.80 ± 0.13^ab^	0.98 ± 0.08^a^
	GC45	23.568	Pentadecane, 4-methyl	2801-87-8	C_16_H_34_	0.31 ± 0.04^ab^	0.28 ± 0.01^b^	0.50 ± 0.10^a^
	GC47	23.882	Pentadecane, 3-methyl-	2882-96-4	C_16_H_34_	0.28 ± 0.03^b^	0.32 ± 0.06^b^	0.48 ± 0.03^a^
	GC72	42.278	Octacosane	630-02-4	C_28_H_58_	0.15 ± 0.02^c^	0.25 ± 0.03^b^	0.36 ± 0.02^a^
Benzenes	GC2	10.577	Benzene,1-ethyl-2,4-dimethyl-	874-41-9	C_10_H_14_	0^c^	0.09 ± 0.01^b^	0.21 ± 0.01^a^
	GC4	12.161	Naphthalene, decahydro-4a-methyl-, trans-	2547-27-5	C_11_H_20_	0^b^	0.36 ± 0.07^a^	0.38 ± 0.06^a^
	GC18	17.698	Naphthalene,1,2,3,4-tetrahydro-1,8-dimethyl-	25419-33-4	C_12_H_16_	0^b^	0.59 ± 0.03^a^	0.84 ± 0.10^a^
Alcohols	GC12	15.857	9-Methyheptacotanol	26741-18-4	C_18_H_38_	0.92 ± 0.11^b^	1.29 ± 0.07^a^	1.28 ± 0.02^a^
	GC41	22.375	2-hexyl-1-decanol	2425-77-6	C_16_H_34_O	0.45 ± 0.05^b^	0.47 ± 0.06^b^	0.66 ± 0.04^a^
	GC71	42.102	1-Docosanol	661-19-8	C_22_H_46_O	0.32 ± 0.07^a^	0.13 ± 0.02^b^	0.18 ± 0.03^ab^
	GC75	46.446	Beta-Sitosterol	83-46-5	C_29_H_50_O	0^b^	0^b^	0.34 ± 0.09^a^
Acids	GC23	18.574	10-METHYLNONADECANE	56862-62-5	C_20_H_42_	1.67 ± 0.06^a^	1.50 ± 0.03^b^	1.52 ± 0.03^ab^
Ethers	GC33	20.918	Di-n-decyl ether	2456-28-2	C_20_H_42_O	1.73 ± 0.11^ab^	1.50 ± 0.05^b^	2.01 ± 0.16^a^
Aldehydes	GC51	27.128	Octadecanal	638-66-4	C_18_H_36_O	0^b^	0.08 ± 0.01^a^	0.11 ± 0.02^a^
	GC68	40.662	1-Pentadecanal	2765-11-9	C_15_H_30_O	0^c^	0.15 ± 0.02^b^	0.43 ± 0.02^a^
Esters	GC53	30.093	Diisobutyl o-phthalate	84-69-5	C_16_H_22_O_4_	0.39 ± 0.01^a^	0.32 ± 0.04^ab^	0.23 ± 0.03^b^
	GC61	36.761	1-Nonadecanol,1-acetate	1577-43-1	C_21_H_42_O_2_	0.36 ± 0.04^b^	0.66 ± 0.16^ab^	0.77 ± 0.07^a^
	GC69	40.939	Octadecanoic acid,2-hydroxyethyl ester	111-60-4	C_20_H_40_O_3_	0^c^	0.15 ± 0.01^b^	0.24 ± 0.02^a^
Amines	GC60	36.184	Hexadecanamide	629-54-9	C_16_H_33_NO	0.23 ± 0.05^b^	0.30 ± 0.03^ab^	0.49 ± 0.10^a^
	GC66	39.771	AKAWAX S-MICROBEADS	124-26-5	C_18_H_37_NO	0.26 ± 0.03^a^	0^b^	0^b^
Phenols	GC67	40.179	2,2-Methylenebis(6-tert-butyl-p-cresol)	119-47-1	C_23_H_32_O_2_	1.63 ± 0.30^c^	2.59 ± 0.14^b^	4.25 ± 0.14^a^

*ID# assigned to each chemical identified via GC-MS; CAS, Chemical Abstracts Service; Peak area% represents mean relative abundance value of three replicates; CK, non-amended; OF, 9000 kg ha^−1^ organic fertilizer (composted cattle manure); BOF, 9,000 kg ha^−1^ biofertilizer (composted cattle manure + Trichoderma inoculum). Data are mean values of three replicates ± standard error (SE). Within a column, values that do not share a letter are significantly different (p < 0.05)*.

**Figure 2:**
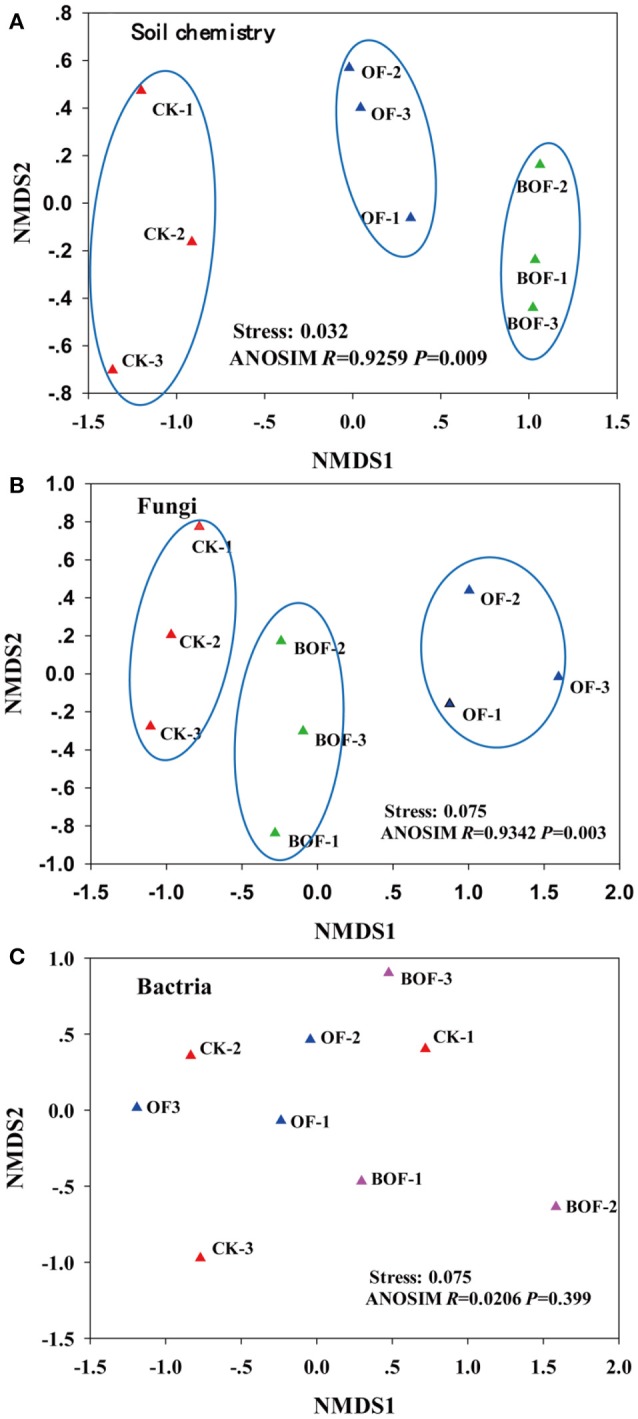
The first two non-metric multidimensional scaling (NMDS) axes of **(A)** soil chemistry community, **(B)** fungal community and **(C)** bacterial community. CK, non-amended; OF: 9,000 kg ha^−1^ organic fertilizer (composted cattle manure); BOF, 9,000 kg ha^−1^ biofertilizer (composted cattle manure + *Trichoderma* inoculum). Each treatment had three replications (CK-1, CK-2, CK-3; OF-1, OF-2, OF-3; BOF-1, BOF-2, BOF-3).

**Figure 3:**
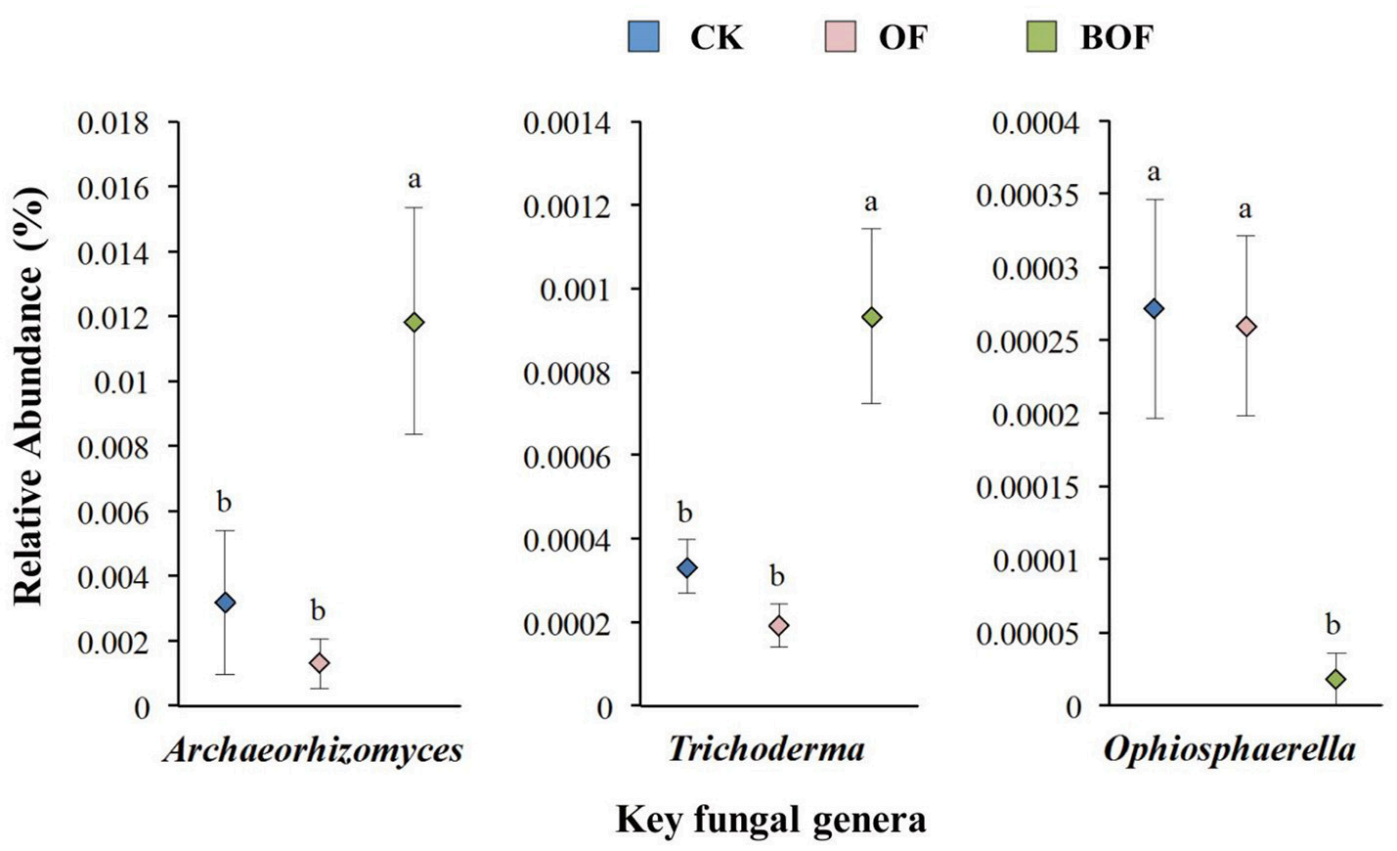
Key fungal genera by fertilization regime. Y-axis scale for figures is based on relative abundance (%) range for different genera. CK (blue): non-amended; OF (pink): 9,000 kg ha^−1^ organic fertilizer (composted cattle manure); BOF (green): 9,000 kg ha^−1^ biofertilizer (composted cattle manure + *Trichoderma* inoculum). Diamonds and lines represent mean values of three replicates ± SE. Values that do not share a letter are significantly different (*p* < 0.05).

Two-dimensional NMDS plots revealed how representative treatments related to differences in soil chemistry and microbial communities (Figure 2). For soil chemistry, pairwise contrasts indicate CK, OF, and BOF plots significantly (*p* < 0.05) separated by axis 1 (Figure 2A). Similarly, fungal communities showed significant (*p* < 0.05) variations by treatment (Figure 2B). However, bacterial communities were relatively similar across all plots (Figure 2C). As for soil microbial community compositions, we observed fewer bacterial genera (4) with significant (*p* < 0.05) differences in relative abundance by treatment compared to fungal genera (14) at the genus level (Table S1). Plots amended with BOF had greater abundance of fungal genera *Archaeorhizomyces* and *Trichoderma* but reduced abundance of *Ophiosphaerella* compared to OF or non-amended plots (Figure 3).

### Soil properties, microbes, and grassland biomass interactions

**Figure 4:**
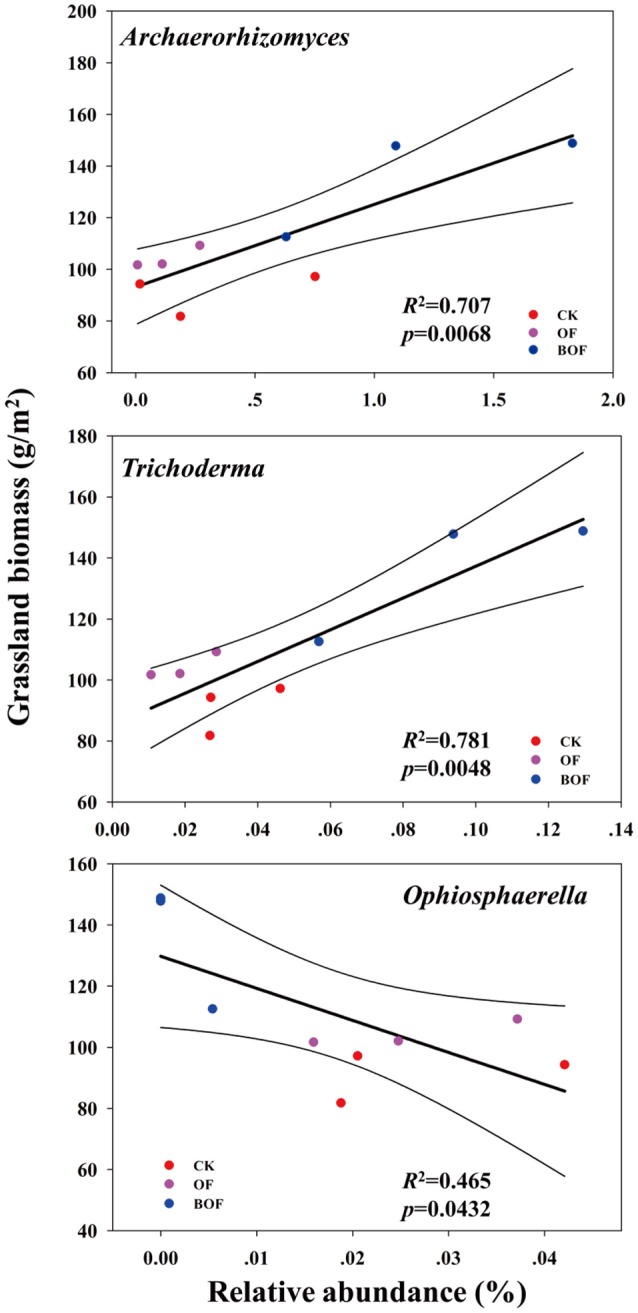
Correlations (*p* < 0.05) between key fungal genera (from Figure 3) and aboveground grassland biomass.

Soil properties differed by fertilization regime (Table S2). Application of any rate of OF or BOF significantly increased soil organic matter and total soil N, compared to non-amended control plots. However, no patterns were observed regarding differences in plant-available P, and there were no significant difference in other soil properties. According to Pearson's correlations (Table 2), the key fungal genera abundances of *Archaeorhizomyces* (*p* = 0.027) and *Trichoderma* (*p* = 0.035) were just positively correlated with higher plant-available P, while *Ophiosphaerella* (*p* = 0.035) was negatively correlated. As shown in Figure 4, positive correlations between grassland biomass and *Archaeorhizomyces* (*p* = 0.0068) and *Trichoderma* (*p* = 0.0048) were found, while *Ophiosphaerella* (*p* = 0.0432) was negatively correlated with aboveground grassland biomass.

**Table 2:** Pearson correlation coefficients between the key fungal genera and soil properties.

**Genus**	**pH**	**OM**	**TN**	**TP**	**TK**	**AN**	**AP**	**AK**
*Archaeorhizomyce*	−0.056	0.152	0.317	0.243	0.188	0.226	0.805^*^	0.588
*Trichoderma*	−0.022	0.204	0.306	0.236	0.151	0.225	0.727^*^	0.564
*Ophiosphaerella*	0.033	−0.246	−0.375	−0.063	−0.008	−0.458	−0.701^*^	−0.627

*OM, organic matter; TN, total N; TP, total P; TK, total K; AN, plant-available N; AP, plant-available P; AK, plant-available K*;

* *indicates significant differences at the 0.05 probability level (Duncan's test)*.

### SEM pathways and grassland biomass

**Figure 5:**
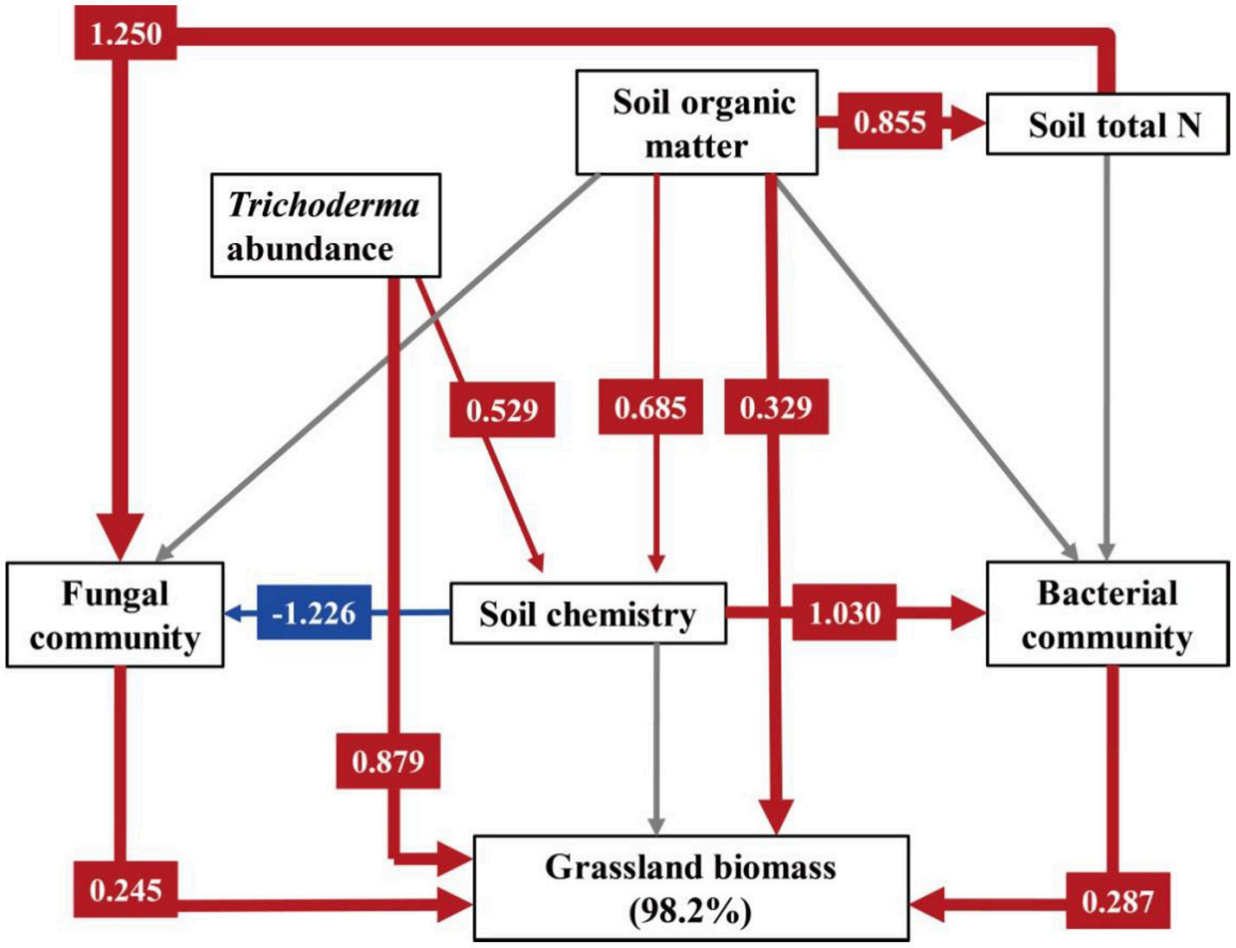
Structural equation modeling (SEM) for grassland biomass. Numbers following variables show the percentage of its variance explained by its predictors. We utilized PCA to demonstrate variations in fungal community across different treatments, and PC1 was selected as the parameter describing the fungal community (PC1 explained 87.25% of total variation in the fungal community). Similarly, PC1 explained 44.85 and 62.77% of total variation in the bacterial community and the soil chemistry, respectively. A path coefficient is analogous to a partial correlation coefficient and describes the strength and sign of the relationship between two variables. Negative pathways are shown as blue lines, positive pathways are shown as red lines, and line thickness represents the intensity of influence. Non-significant pathways are shown in gray. Models are a good fit of our data. Model fits are given in Table S3, and significance levels are provided in Table S4.

Structural equation modeling for grassland biomass was a strong fit with the data (χ^2^ = 4.779, DF = 7, P = 0.687, NFI = 0.946, RFI = 0.838, IFI = 1.027, RMSEA = 0.000, Table S3). As shown in Figure 5, the explanation of the variance in grassland biomass was directly dependent on soil organic matter, *Trichoderma* abundance, bacterial and fungal communities, as affected by fertilization regime. *Trichoderma* abundance had the strongest overall effect (path coefficient = 0.879) on grassland biomass, and the effects of soil organic matter (path coefficient = 0.329), fungal community (path coefficient = 0.245), and bacterial community (path coefficient = 0.287) were also positive. However, the soil chemistry had no significant effect on grassland biomass. Fungal communities were directly mediated by total soil N (path coefficient = 1.250) and the soil chemistry (path coefficient = −1.226). Soil bacterial communities were mainly effected by the soil chemistry (path coefficient = 1.030).

## Discussion

Plant and microbial community structure and biodiversity are crucial for maintaining ecosystem sustainability and productivity (Bell et al., 2005; Cardinale et al., 2006). In our study, we found the application of organic fertilizer or *Trichoderma* biofertilizers resulted in improved aboveground grassland biomass, with optimal results from applying 9,000 kg ha^−1^ *Trichoderma* biofertilizer. Multiple mechanisms may be responsible for increasing grassland biomass. Our results indicate different fertilization regimes drove differences in soil chemistry and edaphic properties, with concomitant shifts in key soil fungal genera. This trend was most pronounced for high inputs of *Trichoderma* biofertilizer.

Differentiation of soil chemistry between selected fertilization regimes (CK, OF, and BOF), presumably formed distinct resource niches in our grassland plots. Many of the identified chemicals were most abundant in plots amended with BOF, compared with CK or OF. Previous studies indicate some of these substances, such as pentadecane and nonadecane, have antifungal properties (Yuan et al., 2012; Raza et al., 2015). In addition, antifungal activity has been reported for the majority of benzenes, alcohols, and phenols (Raza et al., 2015). Based on our findings, we propose antifungal substances were more abundant in the BOF9000 treatment, potentially protecting plants from fungal pathogens. Future studies should focus on how soil chemical profiles influence plant pathogens and could be managed to provide greater plant protection.

Various soil chemical structures are consumed by microbes and can facilitate microbial diversity (Mwafulirwa et al., 2016). We observed a corresponding differentiation of the fungal community but not the bacterial community, based on soil chemistry, driven by fertilization regime. Martin et al. (2015) reported application of compost is a key factor explaining differences in soil microflora. Sun et al. (2015) also demonstrated the role of organic fertilizer in facilitating soil microbial community stability and diversity, which provides a foundation for grassland biomass production. Based on our correlations between microbial taxa and grassland biomass (Figure 5), we propose that *Archaeorhizomyces* and *Trichoderma* may promote plant growth, while *Ophiosphaerella* had the opposite effect. *Trichoderma* spp. are well-known for their capacity to improve plant growth and promote health in agricultural systems (Harman et al., 2004; Gravel et al., 2007; Bae et al., 2009). Examples of these mechanisms include: improving secretion plant stimulatory compounds, such as growth hormones (indole acetic acids, cytokinins, gibberellins, and zeatins; Gravel et al., 2007; Contreras-Cornejo et at., 2009), enhancing solubilization of soil nutrients (Yedidia et al., 2001; Kapri and Tewari, 2010), increasing root length and number of root hairs to absorb nutrients by exploring larger spaces of soil (Bjorkman, 2004; Samolski et al., 2012). The life cycle, ecology, and evolution of *Archaeorhizomycetes* remain largely unknown. However, it is understood that *Archaeorhizomycetes* are non-pathogenic (Rosling et al., 2011). Evidence suggests *Ophiosphaerella* are phytopathogens (Kaminski and Hsiang, 2008). Hciii et al. (2007) reported three *Ophiosphaerella* species (*O. herpotricha, O. korrae, O. narmari*) can cause spring dead spot in Bermudagrass (*Cynodondactylon* [L.] Pers.). Venkatasubbaiah et al. (1994) demonstrated metabolites produced by *O. Herpotricha* can cause necrosis in Bermudagrass and other plant species. *Archaeorhizomyces* and *Trichoderma* were mainly positively correlated with greater plant-available P, while *Ophiosphaerella* presented a negative correlation (Table 2). Taken together, we conclude greater plant-available P was beneficial to aboveground plant biomass by increasing the relative abundances of *Archaeorhizomyces* and *Trichoderma* while decreasing *Ophiosphaerella*. Previous research has reported *Trichoderma* can increase plant P-uptake by increasing P-solubilization in soils (López-Bucio et al., 2015). This increased plant growth was greatest when plant-available P from composted cattle manure was provided along with *Trichoderma* inoculum (BOF). While our current research shows strong correlative linkages between plant, soil, and microbial factors, future research should identify if plant-available P increased plant growth directly, or via indirect effects on the abundance of *Archaeorhizomyces* and *Trichoderma*, and explore methods to better demonstrate a clear mechanistic pathway between microbial community composition, soil chemistry, and grassland biomass.

According to our SEM, soil organic matter, Total N, *Trichoderma* abundance, bacterial community, fungal community, and soil chemistry make a good explain for aboveground plant biomass in meadow steppe grasslands. Christian et al. (2008) demonstrated that fungal communities were most closely associated with changes in soil nutrient status, while soil pH was the best predictor of bacterial communities. This supports our results indicating total soil N had a significant influence on the fungal community and no significant effect on the bacterial community. However, *Trichoderma* had the greatest influence on grassland biomass. This supports the findings of Sivan et al. (1987), who demonstrated plant survival and yield can be increased, following *Trichoderma* inoculation in greenhouse or field settings.

*Trichoderma* biofertilizer (9,000 kg ha^−1^) effectively regulated soil chemistry and microbial communities, driving substantially improved aboveground plant biomass compared to organic fertilizer not containing *Trichoderma*. Certain soil compounds with antifungal activity may ensure individual plant fitness and increase grassland biomass. Plant-available P was beneficial to grassland biomass production, presumably by increasing the relative abundances of beneficial microbes and decreasing phytopathogenic microbes. Soil organic matter, *Trichoderma* abundance, and bacterial and fungal communities have direct influences on grassland biomass, while soil chemistry indirectly increases grassland biomass through alterations in bacterial and fungal communities. Among these factors, *Trichoderma* was the primary contributor to improve grassland biomass. Our study helps to provide a basis for grassland biomass production from the perspective of soil microbial ecology, and may ultimately improve management of grassland soils for key provisioning services. While the selected regimes in our study suggest potential underlying microbial ecological mechanisms, continuous monitoring and analyses of a gradient of fertilization rates will allow a more comprehensive mechanism understanding.

## Author contributions

FZ, GY, and YZ conceived and designed the experiments. FZ and YH performed the experiments. FZ and GL analyzed the data. FZ and YZ contributed reagents, materials, and analysis tools: FZ, AC, JZ, GW, and YZ wrote the paper. All authors reviewed and contributed to the manuscript.

### Conflict of interest statement

The authors declare that the research was conducted in the absence of any commercial or financial relationships that could be construed as a potential conflict of interest.
