# Supplementary material for: Trichoderma Biofertilizer Links to Altered Soil Chemistry, Altered Microbial Communities, and Improved Grassland Biomass
Source: Front Microbiol. 2018 Apr 30;9:848. doi: 10.3389/fmicb.2018.00848 (PMC5937142; doi:10.3389/fmicb.2018.00848)
Supplement: Supplementary file 1 [file Data_Sheet_1.docx]

**Supporting information**

**Fig. S1** (A) Colonial morphology of NAU-18; (B) phylogenetic tree for partial sequences of cloned ITS regions and most closely related fungi. Clones are indicated by their code and accession number (NCBI)


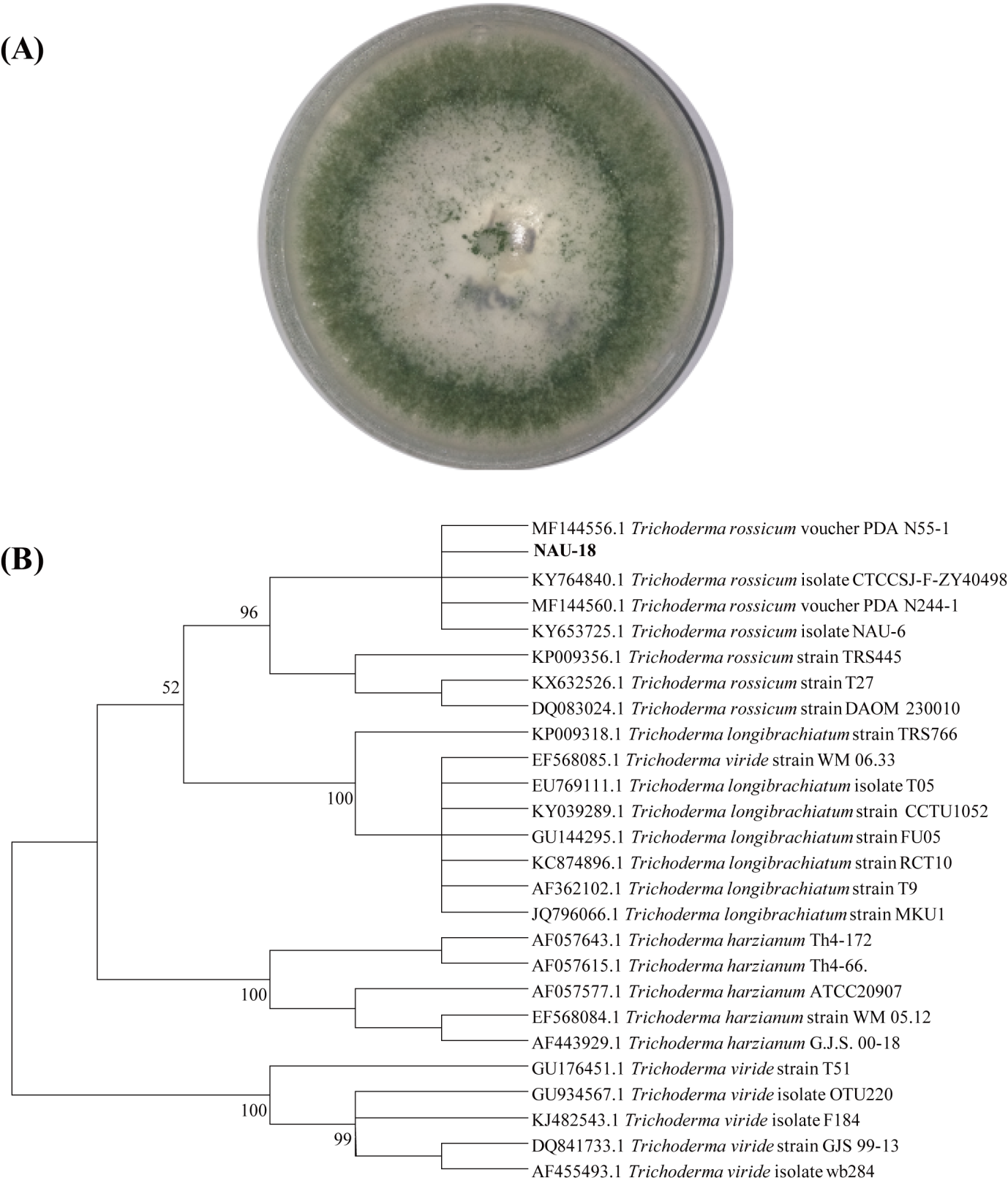


**Fig. S1**

**Table S1** Most abundant bacterial and fungal genera by fertilization regime. CK: non-amended; OF: 9000 kg ha^-1^ organic fertilizer (composted cattle manure); BOF: 9000 kg ha^-1^ *Trichoderma* biofertilizer (composted cattle manure + *Trichoderma* inoculum).

|  | **Genera** | **CK** | **OF** | **BOF** |
| --- | --- | --- | --- | --- |
| **Bacteria** | *Gemmata* | 0.00399a | 0.00324b | 0.00353ab |
|  | *Gemmatirosa* | 0.00309a | 0.00278ab | 0.00230b |
|  | *Friedmanniella* | 0.00085a | 0.00114a | 0.00044b |
|  | *Aquicella* | 0.00087a | 0.00062b | 0.00075ab |
| **Fungi** | *Gliophorus* | 0.02948c | 0.33889a | 0.08000b |
|  | *Pseudogymnoascus* | 0.07282a | 0.02127b | 0.04301b |
|  | *Penicillium* | 0.01473a | 0.00823b | 0.01436a |
|  | *Collembolispora* | 0.00968a | 0.00231b | 0.00545ab |
|  | *Archaeorhizomyces* | 0.00319b | 0.00129b | 0.01184a |
|  | *Exophiala* | 0.00570a | 0.00241b | 0.00249b |
|  | *Periconia* | 0.00273a | 0.00253ab | 0.00187b |
|  | *Simocybe* | 0.00395a | 0.00116b | 0.00053b |
|  | *Oidiodendron* | 0.00286a | 0.00088b | 0.00153ab |
|  | *Rhizophlyctis* | 0.00170a | 0.00033b | 0.00049b |
|  | *Trichoderma* | 0.00033b | 0.00019b | 0.00093a |
|  | *Entrophospora* | 0.00019b | 0.00056a | 0.00018b |
|  | *Cyphellophora* | 0.00051a | 0.00009b | 0.00013b |
|  | *Ophiosphaerella* | 0.00027a | 0.00025a | 0.00001b |

Notes: Data are mean values of three replicates. Within a column, values that do not share a letter are significantly different (*p* < 0.05).

**Table S2** Soil properties by fertilization regime

| **Treatment** | **pH** | **OM (g kg^-1^)** | **TN (g kg^-1^)** | **TP (g kg^-1^)** | **TK (g kg^-1^)** | **AN (mg kg^-1^)** | **AP(mg kg^-1^)** | **AK (mg kg^-1^)** |
| --- | --- | --- | --- | --- | --- | --- | --- | --- |
| Control | 6.63±0.12^a^ | 48.17±3.10^b^ | 2.70±0.10^b^ | 0.60±0.10^a^ | 22.03±4.09^a^ | 210.40±7.15^a^ | 4.20±0.78^ab^ | 167.83±13.31^a^ |
| OF 3000 | 6.80±0.25^a^ | 54.70±3.70^ab^ | 3.07±0.27^ab^ | 0.63±0.09^a^ | 21.63±2.59^a^ | 245.20±14.24^a^ | 4.80±0.36^ab^ | 206.67±17.15^a^ |
| OF 6000 | 6.63±0.03^a^ | 58.40±3.19^a^ | 3.10±0.15^ab^ | 0.63±0.03^a^ | 20.57±2.12^a^ | 246.27±14.81^a^ | 4.23±0.43^ab^ | 182.33±16.54^a^ |
| OF 9000 | 6.80±0.15^a^ | 61.23±1.29^a^ | 3.30±0.06^a^ | 0.67±0.12^a^ | 23.27±2.54^a^ | 220.17±22.83^a^ | 3.33±0.27^b^ | 198.67±5.84^a^ |
| BOF 3000 | 6.40±0.36^a^ | 57.30±1.46^a^ | 3.07±0.12^ab^ | 0.57±0.03^a^ | 21.07±2.77^a^ | 234.53±8.81^a^ | 4.80±0.42^ab^ | 201.83±24.35^a^ |
| BOF 6000 | 6.73±0.03^a^ | 57.60±1.47^a^ | 3.10±0.20^ab^ | 0.67±0.07^a^ | 21.77±1.89^a^ | 232.07±10.20^a^ | 4.17±0.52^ab^ | 182.83±17.98^a^ |
| BOF 9000 | 6.67±0.12^a^ | 62.00±2.19^a^ | 3.27±0.03^a^ | 0.67±0.07^a^ | 21.67±3.82^a^ | 239.77±10.94^a^ | 5.67±0.90^a^ | 222.50±25.87^a^ |

Notes: OM: organic matter; TN: total N; TP: total P; TK: total K; AN: plant-available N; AP: plant-available P; AK: plant-available K; Control: non-amended; OF 3000: 3000 kg ha^-1^ organic fertilizer (composted cattle manure); OF 6000: 6000 kg ha^-1^ organic fertilizer; OF 9000: 9000 kg ha^-1^ organic fertilizer; BOF: 3000 kg ha^-1^ biofertilizer (composted cattle manure + *Trichoderma* inoculum); BOF 6000: 6000 kg ha^-1^ biofertilizer; BOF 9000: 9000 kg ha^-1^ biofertilizer. Data are mean values of three replicates ± standard error (SE). Within a column, values that do not share a letter are significantly different (*p* < 0.05).

**Table S3** Indices of fit for structural equation modelling provided in **Fig. 5**.

| **Fit index** | **χ^2^** | ***P*** | **DF** | **NFI** | **RFI** | **IFI** | **RMSEA** | **AIC** | **ECVI** |
| --- | --- | --- | --- | --- | --- | --- | --- | --- | --- |
| Result | 4.779 | 0.687 | 7 | 0.946 | 0.838 | 1.027 | 0.000 | 60.779 | 7.597 |

Note: χ^2^ = chi-square (minimum function test statistic); DF = degrees of freedom; NFI = normed fit index; RFI = relative fit index; IFI = incremental index of fit; RMSEA = root mean square error of approximation; AIC = Akaike information criteria; ECVI = expected cross-validation index.

**Table S4** Significance levels for the influences of biotic and abiotic factors on grassland biomass, based on structural equation modelling (SEM)

|  |  |  | **Estimate** | **S.E.** | **C.R.** | ***P*** |
| --- | --- | --- | --- | --- | --- | --- |
| Z Total N | <--- | Z Soil organic matter | 0.855 | 0.183 | 4.659 | *** |
| Z Soil chemistry | <--- | Z Soil organic matter | 0.685 | 0.116 | 5.913 | *** |
| Z Soil chemistry | <--- | Z *Trichoderma* abundance | 0.529 | 0.116 | 4.564 | *** |
| Z Fungal community | <--- | Z Total N | 1.250 | 0.258 | 4.837 | *** |
| Z Fungal community | <--- | Z Soil chemistry | -1.226 | 0.220 | -5.566 | *** |
| Z Bacterial community | <--- | Z Soil chemistry | 1.030 | 0.439 | 2.346 | 0.019 |
| Z Bacterial community | <--- | Z Total N | 0.282 | 0.515 | 0.547 | 0.585 |
| Z Bacterial community | <--- | Z Soil organic matter | -0.964 | 0.540 | -1.784 | 0.074 |
| Z Fungal community | <--- | Z Soil organic matter | 0.315 | 0.271 | 1.162 | 0.245 |
| Z Grassland biomass | <--- | Z Soil organic matter | 0.329 | 0.132 | 2.491 | 0.013 |
| Z Grassland biomass | <--- | Z *Trichoderma* abundance | 0.879 | 0.087 | 10.137 | *** |
| Z Grassland biomass | <--- | Z Fungal community | 0.245 | 0.064 | 3.846 | *** |
| Z Grassland biomass | <--- | Z Bacterial community | 0.287 | 0.061 | 4.744 | *** |
| Z Grassland biomass | <--- | Z Soil chemistry | -0.159 | 0.160 | -0.996 | 0.319 |

Notes: Significance. codes: * 0.05, ** 0.01, ***0.001, *p*-values based on 999 permutations
